# Structural Complexity and Informational Transfer in Spatial Log-Gaussian Cox Processes

**DOI:** 10.3390/e23091135

**Published:** 2021-08-31

**Authors:** Adriana Medialdea, José Miguel Angulo, Jorge Mateu

**Affiliations:** 1 Department of Statistics and Operations Research, University of Granada, 18071 Granada, Spain; amedialdea@correo.ugr.es; 2 Department of Mathematics, University Jaume I, 12071 Castellón, Spain; mateu@mat.uji.es

**Keywords:** complexity, divergence, entropy, information transfer, spatial log-Gaussian Cox process

## Abstract

The doubly stochastic mechanism generating the realizations of spatial log-Gaussian Cox processes is empirically assessed in terms of generalized entropy, divergence and complexity measures. The aim is to characterize the contribution to stochasticity from the two phases involved, in relation to the transfer of information from the intensity field to the resulting point pattern, as well as regarding their marginal random structure. A number of scenarios are explored regarding the Matérn model for the covariance of the underlying log-intensity random field. Sensitivity with respect to varying values of the model parameters, as well as of the deformation parameters involved in the generalized informational measures, is analyzed on the basis of regular lattice partitionings. Both a marginal global assessment based on entropy and complexity measures, and a joint local assessment based on divergence and relative complexity measures, are addressed. A Poisson process and a log-Gaussian Cox process with white noise intensity, the first providing an upper bound for entropy, are considered as reference cases. Differences regarding the transfer of structural information from the intensity field to the subsequently generated point patterns, reflected by entropy, divergence and complexity estimates, are discussed according to the specifications considered. In particular, the magnitude of the decrease in marginal entropy estimates between the intensity random fields and the corresponding point patterns quantitatively discriminates the global effect of the additional source of variability involved in the second phase of the double stochasticity.

## 1. Introduction

Log-Gaussian Cox processes define a class of doubly stochastic Poisson processes [1] where the Gaussian intensity-generating function is transformed through exponentiation. These processes (see [2] for a formal definition and properties of log-Gaussian Cox processes) allow the generation of point patterns through a stochastic two-step procedure, where the clustering structure observed in the pattern is due to the inclusion of random heterogeneities in the intensity function. The first applications of these processes are attributed to Coles and Jones [3], who used a log-normal random field as a model of galaxies distribution, and Rathbun [4], who modeled the effect of external variables to describe the patterns formed by the location of organisms. Cox process models fit naturally into the geosciences and ecology fields [5] as the resulting point processes are considered to be driven by environmental variables. There are also contributions in the context of epidemiology [6], ecology [7,8], crime data analysis [9] and seismology [10], among others.

The structural properties of random fields and point patterns can be characterized by means of informational and complexity measures. The concept of entropy, first defined in the context of Information Theory by Shannon [11], and generalized by Rényi [12], as the uncertainty contained in a probability distribution, can be used to quantify the degree of inhomogeneity of each phase of the process. Other informational measures, such as Kullback–Leibler divergence [13] and the corresponding generalization proposed by Rényi [12], are useful to determine the probabilistic local coherence of the phases in the sense of the structural information transferred from the intensity field to the point pattern. A similar analysis can be performed in the context of complexity—for instance, with López-Ruiz, Mancini and Calbet (LMC) measure of complexity [14]. The exponential extension of LMC complexity proposed in [15], although originally introduced to solve the problems that arise for continuous distributions, is also appropriate in the discrete case as it can be interpreted in terms of diversity [16,17]. A related two-parameter generalization, in terms of Rényi entropies of different deformation orders, was formulated by López-Ruiz et al. [18]. Under a similar product-type structure, the two-parameter generalized relative complexity measure introduced by Romera et al. [19], based on Rényi divergences, is used here to describe the local coherence in terms of complexity between the two phases of the doubly stochastic process mechanism.

In the last few decades, since Papangelou’s [20] work defining the entropy rate for continuous point processes in the real line, many other studies have introduced theoretical concepts in the context of Information Theory and complexity for the analysis of point processes. Baratpour et al. [21] assessed the properties of non-homogeneous Poisson processes in terms of entropy; Daley and Vere-Jones [22] extended the definition of entropy for a point process in a *d*-dimensional space, and more recently, Angulo et al. [16] introduced approaches to the analysis of spatial point patterns regarding informational and complexity aspects and focusing on a multifractal context. However, to our knowledge, complexity and information transfer between the two phases of generation of log-Gaussian Cox processes has not been explored.

Many natural phenomena can be modeled by using the family of log-Gaussian Cox processes as the two phases of stochasticity allow us to fit point processes driven, in many cases, by environmental variables. When there is no random field involved, and thus only one phase is considered, we obtain, as a particular case, the family of inhomogeneous Poisson processes. There is a major fundamental difference between these two families and some classical second-order measures cannot clearly distinguish between them. The approach introduced here more deeply considers the system stochastic hierarchical structure, disentangling the two-phase mechanism and analyzing the internal transmission of information, thus highlighting the differences between both types of families. This has a potential effect on a number of applications. For instance, when the point pattern observed is driven in nature by some external environmental variables, this analytical perspective is useful for the assessment of the information that these covariates transfer into the pattern observed. This has immediate applications in crime science, forestry or environmental problems.

We would emphasize at this point that our approach is parallel—complementary, in a certain sense—to a more classical analysis of spatial point patterns based on second-order tools. While, from the latter point of view, we try to detect spatial structure in the pattern, in the novel, former approach, we envisage structural information and its transmission through the different phases defining these processes.

In summary, the main objective of this paper is to analyze the structural transfer of information from the intensity random field to the subsequently generated point pattern in a log-Gaussian Cox process. A marginal global assessment is performed in terms of entropy and complexity measures, and, complementarily, local correspondence is evaluated based on divergence and relative complexity measures. The study, addressed by simulation under a variety of selected scenarios, is primarily focused on sensitivity in relation to the configuration of model parameters, as well as concerning the specification of deformation parameters involved in generalized informational measures.

Section 2 introduces preliminary concepts, both in reference to the class of Cox processes, the object of the present study, and to the information and complexity measures used for structural assessment. The methodological approach and related computational aspects are described in Section 3. The results of the marginal approach and related joint analyses, based on the estimation of information and complexity measures from simulation of the doubly stochastic mechanism, are presented in Section 4, highlighting the most significant aspects. A synthetic discussion in reference to the objectives proposed is provided in Section 5. Concluding remarks, with identification of some relevant open lines for continuing research, are given in Section 6.

## 2. Preliminaries

In this section, we present a summary of the theoretical concepts involved in our analysis. First, we refer to the class of spatial doubly stochastic Poisson processes. Second, we review the definitions and basic properties of some well-known information measures, as well as related complexity measures developed in the last few decades.

### 2.1. Log-Gaussian Cox Processes

Cox processes [2], called doubly stochastic Poisson processes, constitute an important class of spatial point pattern models useful for the representation of a rich variety of structural point dependency effects. In essence, a Cox process can be defined as a Poisson process with a random intensity function, which can be technically formalized in terms of a hierarchical two-step procedure: first, a non-negative random field $\Lambda \left(x\right)$ is generated on a given continuous domain $D\subseteq R^{2}$; second, for the obtained realization $\lambda \left(x\right)$, a Poisson process with intensity function $\lambda \left(x\right)$ is built. For $\Lambda \left(x\right)$ to be valid, it is required that each realization is integrable on bounded sets. This mechanism allows the incorporation of heterogeneities of an intrinsic random nature at the intensity level. Cox processes are widely used in practice due to their meaningful and practical construction.

In many applications, it is appropriate to assume that the intensity generating random field can be modeled as a suitable function of a Gaussian random field. Since the probabilistic structure can be completely specified in terms of the first- and second-order moments of the latter, consequently, this assumption represents some advantages regarding inferential aspects and interpretations.

Under this approach, research has been particularly focused on the class of log-Gaussian Cox processes, for which the intensity-generating random function is defined as

$$\Lambda \left(x\right)=\exp\left(G\left(x\right)\right),$$

where $G\left(x\right)$ is a Gaussian random field, with the first- and second-order moments expressed, respectively, as

$$\mu \left(x\right)=E\left[G\left(x\right)\right],$$

$$C\left(x,y\right)=Cov\left(G\left(x\right),G\left(y\right)\right)=E\left[G\left(x\right)G\left(y\right)\right]-\mu \left(x\right)\mu \left(y\right).$$

In this paper, the widely used Matérn class [23,24,25,26] is considered as the covariance model for the intensity-generating Gaussian random field, due to its high flexibility and richness for the representation of a wide variety of stationary spatial dependence scenarios.

This model is defined by the homogeneous and isotropic covariance function

$$K\left(r\right)=\frac{\sigma^{2}}{2^{\nu -1}\Gamma \left(\nu \right)}{\left(\frac{2\nu^{1/2}r}{\rho }\right)}^{\nu }K_{\nu }\left(\frac{2\nu^{1/2}r}{\rho }\right),$$

where $K_{\nu }$ is the modified Bessel function of the second kind, $\sigma^{2}\geq 0$ is the variance of the Gaussian random field, and ν>0 and ρ>0, respectively, represent smoothness and scale parameters.

### 2.2. Information and Complexity

Information Theory arose in the context of Communication Theory for solving the emerging problems in message transmission through noisy channels. Based on the seminal concept of ‘information content’ introduced by Hartley [27], as a measure of the amount of information provided by the knowledge of the state in a finite system, Shannon [11] formulated ‘entropy’ (or ‘information entropy’) as a measure of the uncertainty intrinsic to a given discrete probability distribution, $p=(p_{1},\cdots ,p_{N})$, in terms of the expectation

$$H\left(p\right):=-E\left[\ln\left(p\right)\right]=-\sum_{i=1}^{N}p_{i}\ln\left(p_{i}\right)$$

(i.e., the expected information content). Shannon entropy is maximum for a system consisting of *N* equiprobable states, with $H_{max}=\ln N$. The reciprocal value given by the difference $H_{max}-H\left(p\right)$, generally normalized dividing by $H_{max}$, is interpreted as ‘redundancy’ ([11]).

Among various well-known generalizations, formally derived by a certain relaxation of the intrinsic axiomatic, Rényi [12] entropy of order α (with α being a ‘deformation parameter’ on the probability distribution p) is defined by the expression

$$H_{\alpha }\left(p\right):=\frac{1}{1-\alpha }\ln\left(\sum_{i=1}^{N}p_{i}^{\alpha }\right)=\frac{1}{1-\alpha }\ln\left(E[p^{\alpha -1}]\right)\;\left(\alpha \neq 1\right).$$

This entropy is a decreasing function of the order α, and Shannon entropy is obtained as the limiting case for α→1 (hence being also denoted as $H_{1}\left(p\right)$). Rényi entropy is also maximum, for any α, in the case of equiprobability, again with $H_{\alpha ,max}=\ln N$. Correspondingly, the difference $H_{max}-H\left(p\right)$, or its normalization dividing by $H_{max}$, is interpreted as ‘redundancy of order α’. The exponential of Rényi entropy (in particular, Shannon entropy) can be seen as a ‘diversity index of order α’ representing, in a certain sense, the intrinsic number of states of the system according to the reference distribution ([17]).

The meaning and added value of Rényi entropy with respect to Shannon entropy is better understood in relation to the α-power distortion implied on the argument distribution. In particular, for α>1, in a non-equilibrium distribution, increasing values of α tend to lead to higher probabilities, in a sensitive way that depends on the whole internal structure of the distribution. Conversely, for α<1, decreasing α tends to equilibrate, in a certain way, as mentioned before, the reference distribution. Thus, the curve of Rényi entropies can be used for assessing and comparing systems that even may have equal Shannon entropy.

Another important concept for the structural assessment of a random system is complexity, which, among other approaches introduced in the literature, has been specifically understood, in a probabilistic informational sense, as a departure from both degeneracy into one single state (‘perfect order’) and equiprobability (‘complete disorder’). In this direction, López-Ruiz et al. [14] proposed the following formulation of a complexity measure (usually referred to as the ‘LMC complexity’): for a given discrete probability distribution p,

$$C_{LMC}\left(p\right)=H\left(p\right)\cdot D\left(p\right),$$

with the first factor being the Shannon entropy and the second one representing the disequilibrium defined as the quadratic distance

$$D\left(p\right)=\sum_{i=1}^{N}{\left(p_{i}-\frac{1}{N}\right)}^{2}.$$

We may remark at this point that, as occurs with most proposals of complexity measures, the widely used product-type formalism, and particularly the LMC complexity measure, has certain inherent limitations, and its interpretation as a quantifier of some specific aspects within the broad and multidimensional concept of ‘complexity’ must be restricted to the relative balance between the two factors involved (see, for instance, the critical discussion by [28]).

For the case of continuous probability distributions, Catalán et al. [15] proposed a modified ‘exponential’ version of the LMC complexity (in the sense that Shannon entropy is replaced with its exponential), which was the basis for the formulation of a two-parameter generalized complexity measure, proposed later by López-Ruiz et al. [18]. In fact, the latter is perfectly meaningful also in the discrete case (see [16], for instance, in relation to [17] diversity index), for which it takes the form

(1) $$C_{\alpha ,\beta }\left(p\right):=e^{H_{\alpha }\left(p\right)-H_{\beta }\left(p\right)},\;\mathrm{with}\;0<\alpha ,\beta <\infty .$$

Therefore, this complexity measure quantifies, in the exponential scale (interpreted as diversity, as mentioned before), the sensitivity of the argument distribution to power distortion in terms of the increments of the Rényi entropy curve between two given values, α and β, of the deformation parameter. A comprehensive display is usually given in the form of an (α,β)-map, for selected deformation parameter ranges.

While entropy and complexity measures enable a comparison in global terms (marginally) of two given probability distributions, a proper assessment of their structural (state by-state) dissimilarity, or lack of mutual coherence, is achieved by means of divergence and relative complexity measures. For two given probability distributions $p=(p_{1},\cdots ,p_{N})$ and $q=(q_{1},\cdots ,q_{N})$ on a system with *N* possible states, Kullback and Leibler [13] defined the divergence of p from q as

$$\begin{array}{cc} KL(p∥q) & :=\sum_{i=1}^{N}p_{i}\ln\left(\frac{p_{i}}{q_{i}}\right)=E_{p}\left[\ln\left(\frac{p}{q}\right)\right]. \end{array}$$

This is a non-symmetric (‘directed’), non-negative measure, vanishing if and only if p=q. A corresponding generalization based on a ‘deformation parameter’ is also given by Rényi [12] divergence of order α of p from q, defined as

$$\begin{array}{cc} H_{\alpha }\left(p∥q\right) & :=\frac{1}{\alpha -1}\ln\left(\sum_{i=1}^{N}p_{i}{\left(\frac{p_{i}}{q_{i}}\right)}^{\alpha -1}\right)\\ \; & =\frac{1}{\alpha -1}\ln\left(E_{p}\left[{\left(\frac{p}{q}\right)}^{\alpha -1}\right]\right)\;\left(\alpha \neq 1\right). \end{array}$$

For fixed argument distributions, Rényi divergence is a non-decreasing function of the deformation parameter. For α→1, $H_{\alpha }\left(p∥q\right)$ tends to KL(p∥q) (hence with the latter being also denoted as $H_{1}\left(p∥q\right)$). The exponential of Rényi divergence (in particular, Kullback–Leibler divergence) can be interpreted as a ‘relative diversity index of order α’ (see [16]).

In the special case of the divergence of order α from equiprobability, i.e., with $q\equiv \left[\frac{1}{N}\right]$, its value can be calculated as

$$H_{\alpha }\left(p∥\left[\frac{1}{N}\right]\right)=H_{\alpha ,max}-H_{\alpha }\left(p\right)=\ln N-H_{\alpha }\left(p\right),$$

hence being known as the ‘information difference’ of order α for p.

A divergence-based formulation of a product-type generalized relative complexity measure was introduced for continuous distributions by Romera et al. [19]. In the discrete case, it takes the form (see Angulo et al. [16], in relation to a concept of ‘relative diversity’)

(2) $$C_{\alpha ,\beta }\left(p∥q\right):=e^{H_{\alpha }\left(p∥q\right)-H_{\beta }\left(p∥q\right)},\;with\;0<\alpha ,\beta <\infty .$$

In particular, for $q\equiv \left[\frac{1}{N}\right]$, the generalized complexity and relative complexity measures (1) and (2) are reciprocal in the following sense:

$$C_{\alpha ,\beta }\left(p∥\left[\frac{1}{N}\right]\right)=\frac{1}{C_{\alpha ,\beta }\left(p\right)}=C_{\beta ,\alpha }\left(p\right).$$

Both for Rényi divergence and generalized relative complexity, the implications of the deformation parameter are directly related (similarly as mentioned for the cases of Rényi entropy and generalized complexity) to the power distortion effect derived on the two distributions involved; that is, the curve of Rényi divergences, and the corresponding map of generalized relative complexities, provide information about the sensitivity of divergence (as the directed distance between two distributions on a given system), or relative diversity in the exponential scale, for different parameter deformation values.

A synthetic review, providing connective relations and interpretation of the above-summarized information complexity concepts, is given by Angulo et al. [16].

## 3. Methodology

As mentioned in Section 1, the analysis is aimed at characterizing, both in a global (marginal) and a local sense, the information transfer from the intensity field to the point pattern. To this end, an empirical approach based on the lattice box-counting methodology is adopted.

Formally, the analysis is based on the simulation of log-Gaussian Cox processes for different scenarios, under different varying configurations of the covariance function and mean parameters of the intensity-generating Gaussian random field; see details in Table 1. For each specific configuration, in the first stage, *M* independent replicates of the intensity field are simulated on the square $D={\left[0,10\right]}^{2}$, based on a 180×180 pixel window. From each realization, in the second stage, one or multiple point patterns are independently generated, as discussed below according to the objective of the analysis.

**Figure 1:**
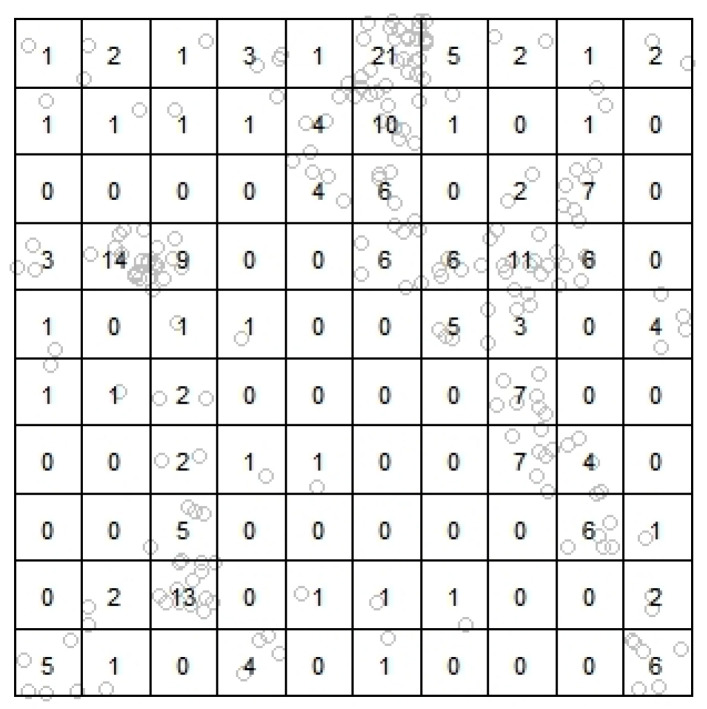
Example of quadrat-based counts of a point pattern.

For assessment, the information complexity measures are applied considering, in particular, a 10×10 lattice overlaid on the domain *D*. The choice of this lattice resolution has been experimentally established with the aim of preserving a certain balance based on the cell size fixed, being large enough to reflect a spatial distribution coherent with the point pattern structure and, at the same time, adequately small regarding the smoothing effect derived on the intensity field realization (see, for example, Figure 1).

**Figure 2:**
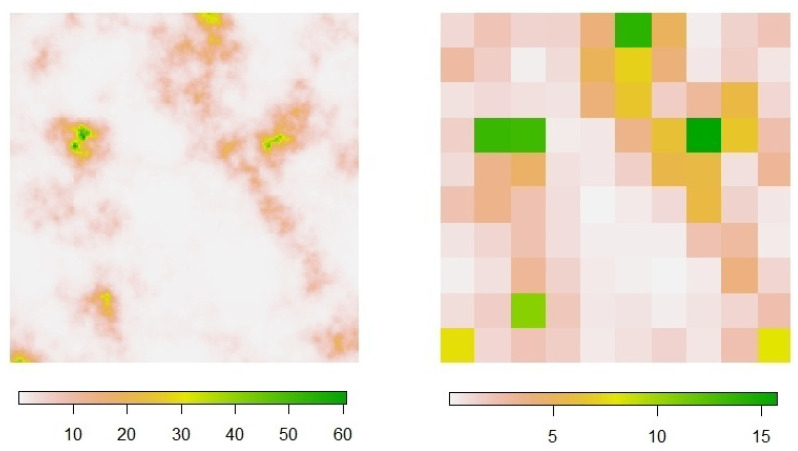
Example of intensity field realization **(left**) and derived mean intensity raster (**right**).

Each intensity realization, λ[m] (for m=1,⋯,M), is converted to a quadrat-based (‘raster’) mean intensity field by averaging its values within each cell (as displayed in Figure 2), and further transformed by normalization into a discrete probability distribution, denoted as $q\left[m\right]=(q{\left[m\right]}_{i,j}:i,j=1,\cdots ,10)$, hence representing the relative intensity for the quadrat design adopted; in the study performed here and in Section 4, we take M=100. As for the point patterns, the relative frequencies obtained from intra-cell event counting provide the quadrat-based reference discrete probability distribution, denoted as $p\left[m\right]=(p{\left[m\right]}_{i,j}:i,j=1,\cdots ,10)$ for just one correspondingly generated pattern, or $p\left[m;r\right]=(p{\left[m;r\right]}_{i,j}:i,j=1,\cdots ,10)$, with r=1,⋯,R, in the case of multiple patterns.

Entropy (Shannon, Rényi) and complexity (LMC, generalized) measures are marginally evaluated on the related probability distributions derived, for the assessment regarding the characterization and global transfer of information between the two phases of stochasticity. In particular, according to the scenarios considered, the analysis of results is focused on sensitivity with respect to variations in the model and deformation parameters.

Additionally, the degree of local (cell-based) coherence between the distributions corresponding to the two phases is analyzed on the basis of divergence (Kullback–Leibler, Rényi) and relative complexity (generalized) measures.

**Figure 3:**
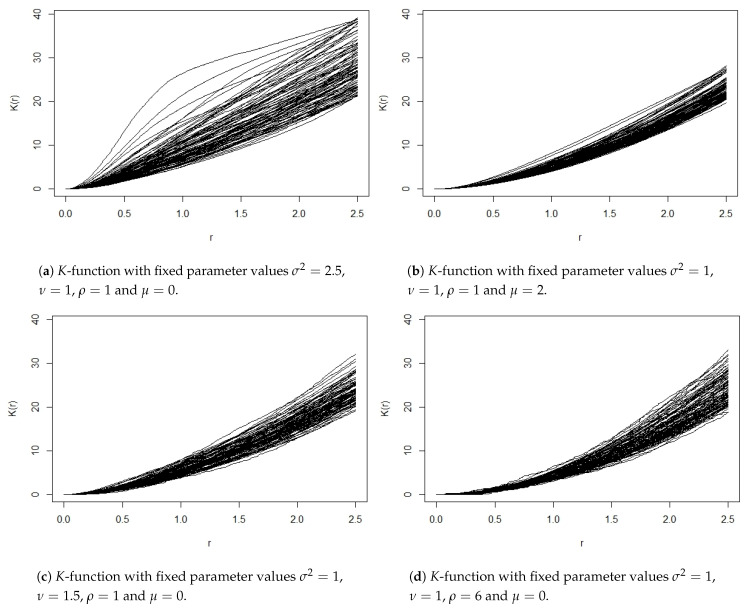
*K*-function of 100 point patterns of log-Gaussian Cox processes generated under different scenarios.

The *K*-function plots displayed in Figure 3 show the effect of diverse settings of the covariance parameters in the spatial correlation of each pattern. Analysis based on information complexity measures is expected to reflect, according to model specifications, significant structural features in the internal hierarchical construction of the processes.

As mentioned, from each simulation of the intensity field, either one or multiple independent point pattern replicates can be analyzed. The second strategy specifically seeks to discriminate the relative contribution from the two phases of stochasticity to the structural variability of the process. For a preliminary assessment regarding this issue, R=100 independent patterns are generated from each one of M=100 replicates of the intensity random field (hence having in total M×R=10,000 patterns), for varying values of the variance parameter according to Scenario 1 (see Table 1). Different standard deviation values of Shannon entropy and Kullback–Leibler divergence, summarized in Table 2 and Table 3, respectively, are computed for comparison as follows:

- ‘Mean Intra SD’—The average of the internal standard deviations of the entropy and divergence values obtained within each of the *M* sets of *R* point patterns:
  $$\underset{\left(m\right)}{Ave}\left(\underset{\left(r\right)}{SD}\left(H(p[m;r])\right)\right),\;\underset{\left(m\right)}{Ave}\left(\underset{\left(r\right)}{SD}\left(KL(p[m;r]∥q[m])\right)\right)$$
- ‘Inter-Mean SD’—The standard deviation of the internal average entropy and divergence values obtained within each of the *M* sets of point patterns:
  $$\underset{\left(m\right)}{SD}\left(\underset{\left(r\right)}{Ave}\left(H(p[m;r])\right)\right),\;\underset{\left(m\right)}{SD}\left(\underset{\left(r\right)}{Ave}\left(KL(p[m;r]∥q[m])\right)\right)$$
- ‘Total SD’—The standard deviation of the entropy and divergence values obtained for the M×R patterns:
  $$\underset{\left(m,r\right)}{SD}\left(H(p[m;r])\right),\;\underset{\left(m,r\right)}{SD}\left(KL(p[m;r]∥q[m])\right)$$
- ‘Inter-Single SD’—The standard deviation of entropy and divergence values based on only one single pattern being generated from each of the *M* replicates of the intensity field:
  $$\underset{\left(m\right)}{SD}\left(H(p[m])\right),\;\underset{\left(m\right)}{SD}\left(KL(p[m]∥q[m])\right)$$

(In the expressions above, ‘Ave’ and ’SD’ denote the usual basic operations given, for a generic set of real numbers $\left\{x_{l}:l=1,\cdots ,L\right\}$, by $\underset{\left(l\right)}{Ave}\left(x_{l}\right)=\frac{1}{L}\sum_{l=1}^{L}x_{l}$ and $\underset{\left(l\right)}{SD}\left(x_{l}\right)={\left\{\frac{1}{L}\sum_{l=1}^{L}{\left(x_{l}-\underset{\left(l^{\prime }\right)}{Ave}\left(x_{l^{\prime }}\right)\right)}^{2}\right\}}^{\frac{1}{2}}$. Clearly, in the special case where R=1, the ‘Mean Intra SD’ is null and the three remaining quantities, ‘Inter-Mean SD’, ‘Total SD’ and ‘Inter-Single SD’, become equivalent).

In general terms, comparing the results for ‘Total SD’ and ‘Inter-Single SD’, we can see that the total variability of the entropy and divergence measures obtained from multiple patterns can be well captured with just a single pattern generated from each realization of the intensity field. Therefore, in the analyses performed in Section 4, we simulate patterns without replicates.

**Figure 4:**
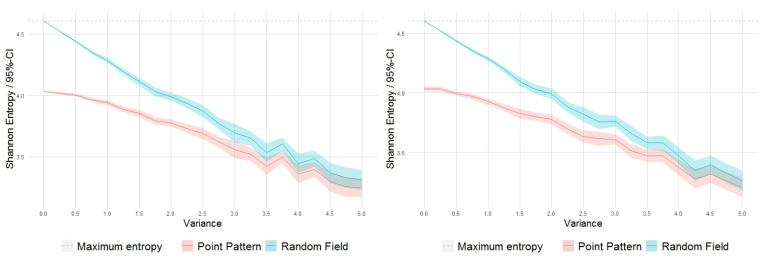
Mean Shannon entropy values for point patterns obtained with 100 replicates (**left**) and with only one generation (**right**) from each intensity random field realization.

**Figure 5:**
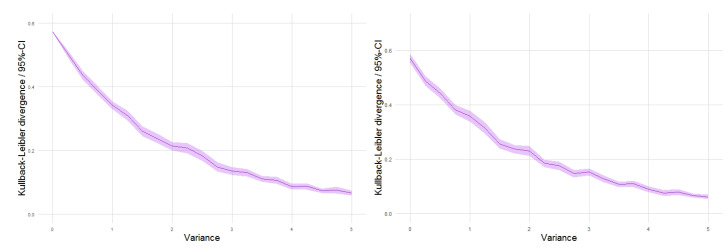
Mean Kullback–Leibler divergence values for point patterns obtained with 100 replicates (**left**) and with only one generation (**right**) from each intensity random field realization.

Nevertheless, from the ‘inter-intra’ analysis based on multiple replicates, it is interesting to observe that, as the variance parameter $\sigma^{2}$ increases, the average internal variability of entropy and divergence values for the patterns derived from each intensity realization progressively decreases compared to a significant increase in the corresponding variability for the intra-averaged entropy values, and a more stable behavior for the divergence values. This is also visualized in the 95% confidence-level bands in the curves represented in the plots of Figure 4 and Figure 5, respectively, for the mean entropy and mean divergence values obtained with and without multiple replicates.

With the aim of providing a benchmark case, 100 independent realizations of a homogeneous Poisson process with constant intensity λ=10, and similarly for a log-Gaussian Cox process with (pixel-based) white noise intensity, are simulated and analyzed. In the second case, the intensity generating the Gaussian random field has a local normal distribution with mean μ=0 and variance $\sigma^{2}=2\ln10$, generating an average intensity close to 10. These cases result in patterns with low structuring, establishing certain upper and lower bounds for entropy and complexity, respectively.

The study is carried out using R software—in particular, the spastat and RandomFields packages for simulating the processes using the rLGCP function. The raster package is used for discretization of the intensity random field.

## 4. Analysis

### 4.1. Weak Structure Reference Processes

**Figure 6:**
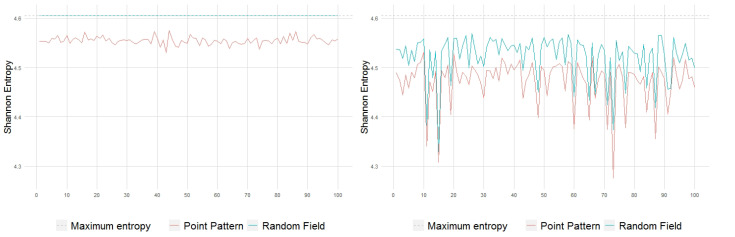
Shannon entropy of 100 point patterns and their generating intensity field, with constant λ=10 (**left**) and with white noise-based intensity (**right**).

**Figure 7:**
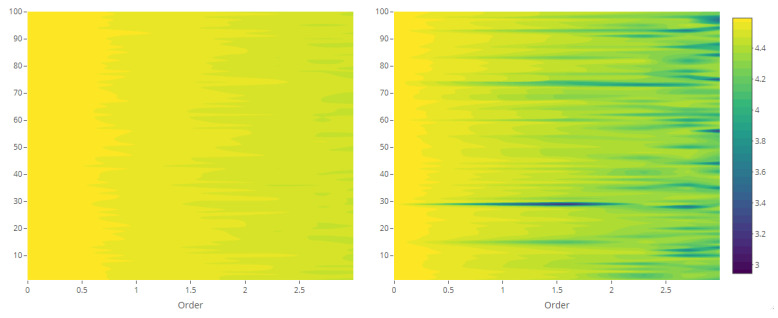
Rényi entropy of 100 point patterns and their generating intensity field, with constant λ=10 (**left**) and with white noise-based intensity (**right**).

Due to the trivial structure of the reference homogeneous and white noise intensity-based Poisson processes, significant inhibition or aggregation effects are not expected to be observed in the pattern realizations, i.e., points should be mostly evenly distributed throughout the spatial domain; as a consequence, Shannon and Rényi entropies (Figure 6 and Figure 7) take high values, still showing a certain global gain in structuring, slightly increased in the white noise intensity case. Obviously, as the intensity field is constant for the homogeneous Poisson process, its quadrat-based distribution is perfectly uniform and entropy reaches the maximum possible value $H_{\alpha ,max}=\ln\left(10^{2}\right)=4.60517$, for all α, whilst the entropy of the quadrat-based distribution for the white noise intensity is always close to this maximum.

**Figure 8:**
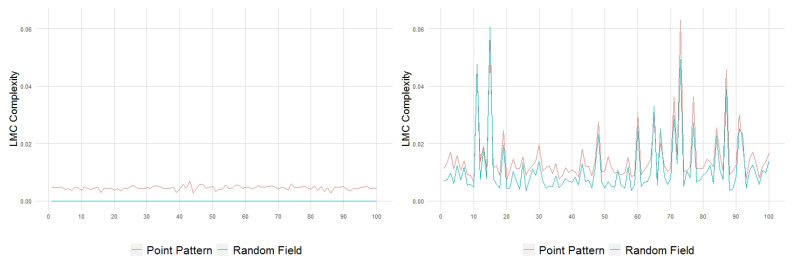
LMC complexity of 100 point patterns and their generating intensity field, with constant λ=10 (**left**) and with white noise-based intensity (**right**).

On the other hand, the LMC complexity of the pattern realizations, in both cases, is close to the minimum value 0; see Figure 8. This is expected since $H∼H_{max}$ and D∼0. However, in terms of this complexity measure, the pattern structuring is still slightly higher in the white noise intensity case.

**Figure 9:**
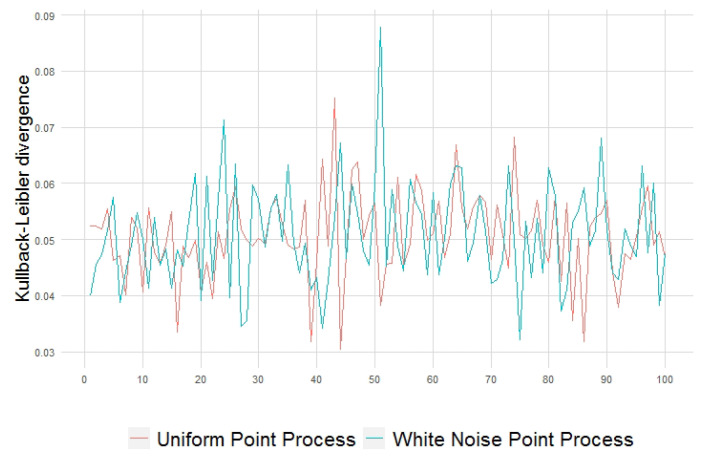
Kullback–Leibler divergence between 100 point patterns and their generating intensity field, with constant λ=10 (**red**) and with white noise-based intensity (**blue**).

**Figure 10:**
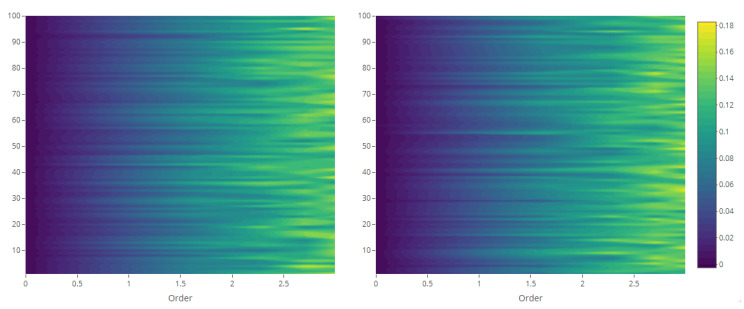
Rényi divergence between 100 point patterns and their generating intensity field, with constant λ=10 (**left**) and with white noise-based intensity (**right**).

**Figure 11:**
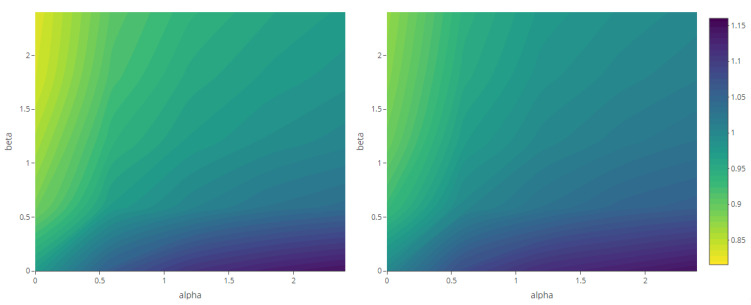
Generalized relative complexity between 100 point patterns and their generating intensity field, with constant λ=10 (**left**) and with white noise-based intensity (**right**).

The structuring effect from the additional source of variability present in the generation of the point patterns is also assessed by the local discrepancies measured between the point pattern and intensity quadrat-based distributions, as shown in Figure 9 for Kullback–Leibler divergence, Figure 10 for Rényi divergence, and Figure 11 in terms of the generalized relative complexity. The initial variability present in the white noise intensity field is reflected as well in the generation of the patterns.

### 4.2. Marginal Analysis

**Figure 12:**
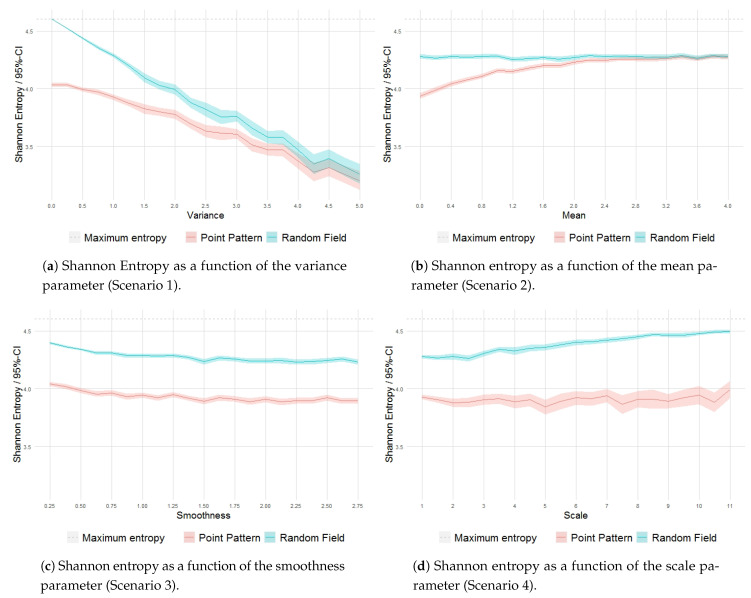
Shannon entropy as a function of the variance, mean, smoothness and scale parameters.

The behavior of entropy is highly influenced by the isolated variation in each parameter. The increase in the variance parameter results in clusters with a high concentration of points, which implies a decrease in entropy values as the distributions move further away from equiprobability (Figure 12a). Large values of the variance parameter increase the local variability in the magnitude of clustering.

The increase in the mean parameter is reflected in a larger mean intensity of the process, which results in an exponential increase in the mean number of points in the pattern. As a result, while the entropy values of the quadrat-based probability distribution from the intensity field essentially remain at a constant level below $H_{max}$ (related to the specification of a fixed variance $\sigma^{2}=1$), the entropy values for the corresponding patterns progressively tend to increase from below this level (Figure 12b).

The ν parameter controls the differentiability of the intensity field and determines its smoothness. In Figure 12c, the entropies of the two phases of the processes show a slight gradual decrease as the parameter increases, while the differences between them remain steady. This is partly influenced by the adopted box size, taking into account the local nature of the smoothness effect.

The scale parameter ρ measures the decay of covariance in the intensity field as a function of the distance. The covariance decays more slowly as ρ increases, which results in more homogeneous fields, limiting the formation of clusters. As a result, larger values of entropy are obtained for the intensity fields as the scale parameter increases, whilst, for the point patterns, we can observe an increase in the variability of the measure (Figure 12d).

**Figure 13:**
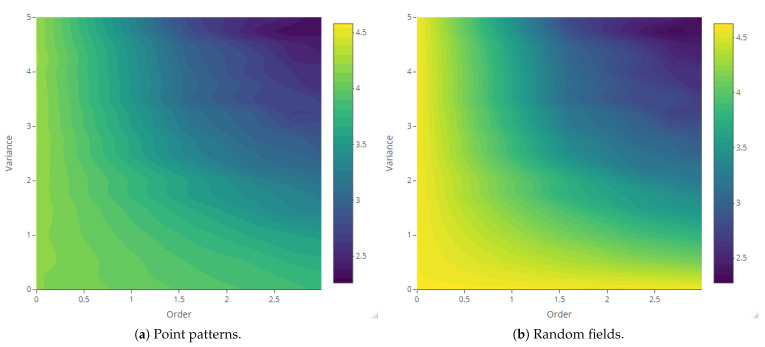
Rényi entropy of log-Gaussian Cox processes as a function of the variance parameter (Scenario 1) and order α={0,ln5,ln10,ln15,ln20}.

**Figure 14:**
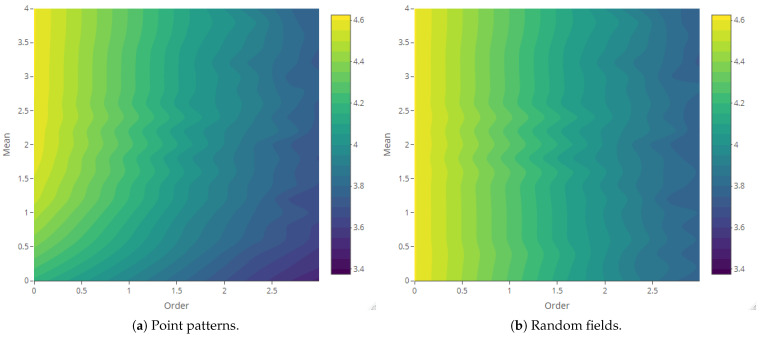
Rényi entropy of log-Gaussian Cox processes as a function of the mean parameter (Scenario 2) and order α={0,ln5,ln10,ln15,ln20}.

**Figure 15:**
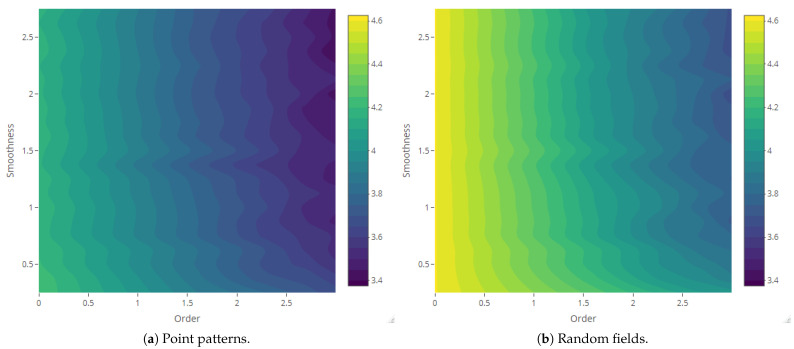
Rényi entropy of log-Gaussian Cox processes as a function of the smoothness parameter (Scenario 3) and order α={0,ln5,ln10,ln15,ln20}.

**Figure 16:**
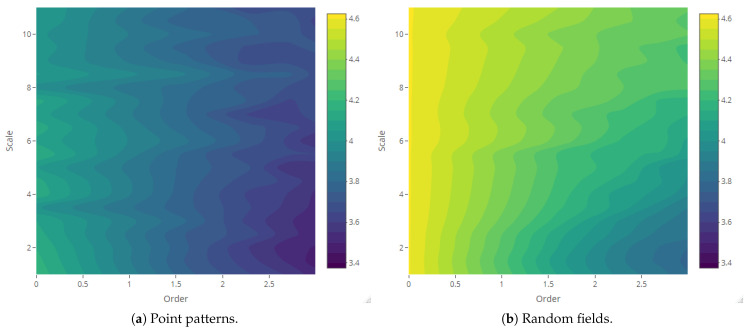
Rényi entropy of log-Gaussian Cox processes as a function of the scale parameter (Scenario 4) and order α={0,ln5,ln10,ln15,ln20}.

The maps of Rényi entropy (Figure 13, Figure 14, Figure 15 and Figure 16) show the evolution of the global heterogeneity in each scenario for different magnitudes of the deformation parameter, which produces a distortion effect on the probability distributions.

**Figure 17:**
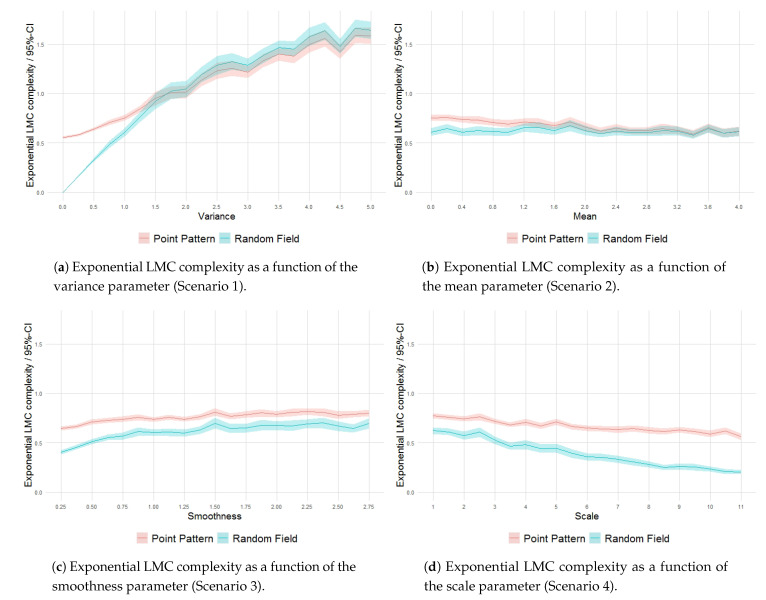
Exponential LMC complexity as a function of the variance, mean, smoothness and scale parameters.

The behavior of the processes in terms of the exponential LMC complexity (Figure 17) reflects, in general, an increase in structuring from the intensity field to the generated point pattern (the inversion observed in Figure 17a in relation to the variance parameter is related to the quadrat-counting procedure and the low mean value specified).

**Figure 18:**
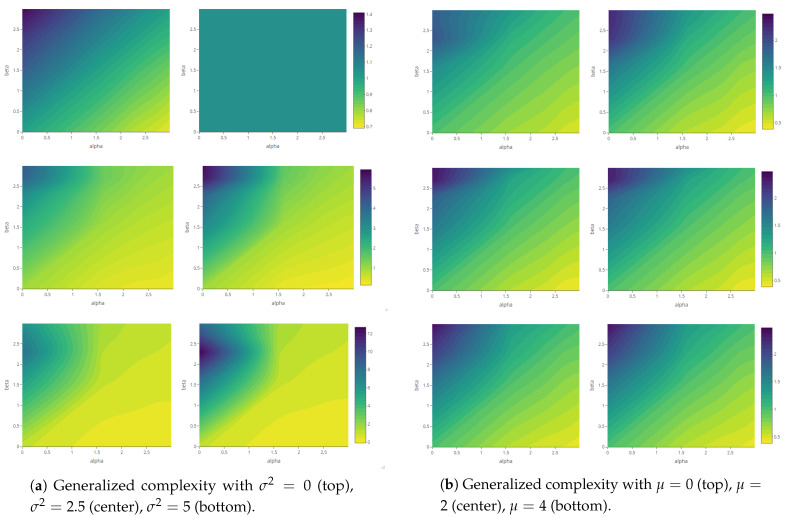

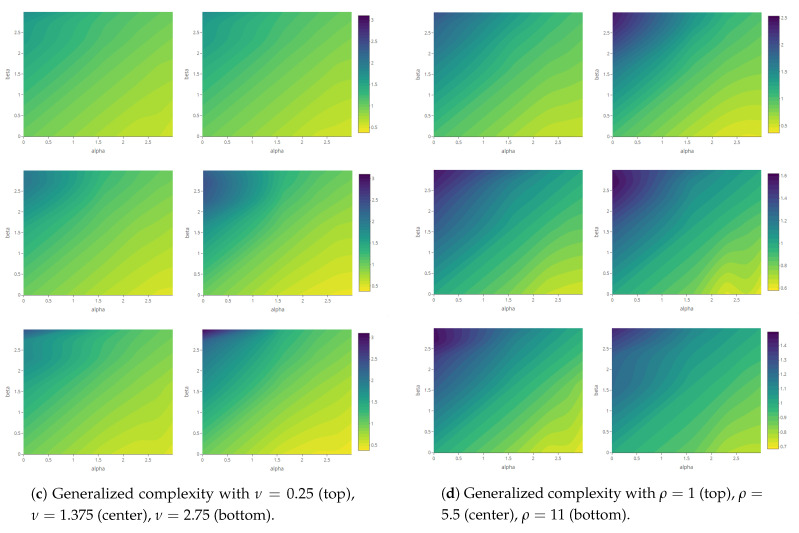
Generalized complexity as a function of deformation parameters α and β (with fixed parameter values according to each scenario—see Table 1).

The maps of the generalized complexity (Figure 18) show the structural variation in each system for different combinations of the deformation parameters α and β.

### 4.3. Joint Analysis

In this section, we focus on the divergence-based measures that allow the local comparison of two probability distributions in each possible state of the system. In particular, we aim to compare the distributions of the intensity fields and the point patterns to asses the state-by-state information transfer, and hence structural contribution, between the two phases.

**Figure 19:**
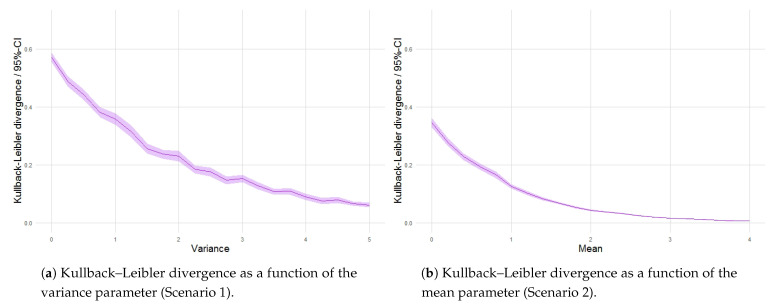

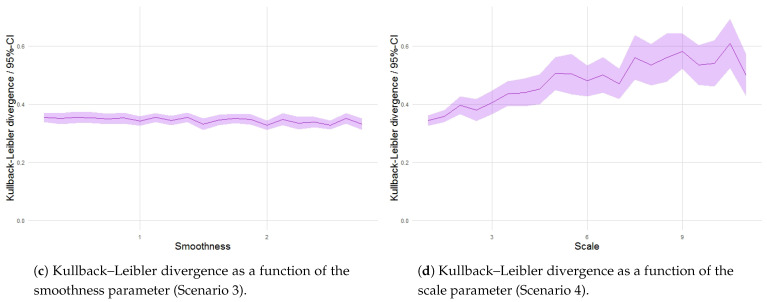
Kullback–Leibler divergence between point patterns and intensity random fields as a function of the variance, mean, smoothness and scale parameters.

As we have seen in the previous section, Shannon entropy for the distributions of intensity fields and corresponding point patterns in Scenarios 1 and 2 (Figure 12a,b) converges globally as the variance and mean parameter values increase, respectively. The Kullback–Leibler divergence shows that there exists as well increasing local coherence in the transfer of information (Figure 19a,b). On the other hand, in Scenario 3, divergence essentially remains steady with respect to changes in the smoothness parameter (Figure 19c), which suggests that, under the quadrat-based approach adopted, the structural transfer is not overly sensitive to variations in the smoothness parameter. In Scenario 4, the loss of the structure in the intensities induced by high magnitudes of the scale parameter increases the stochasticity in the generation of the patterns in the second phase (Figure 19d).

**Figure 20:**
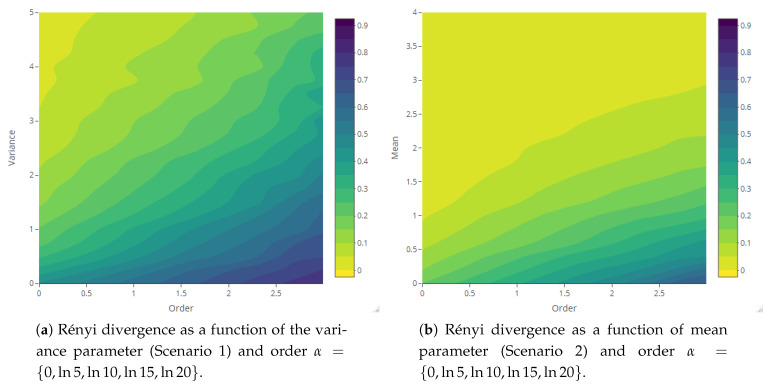

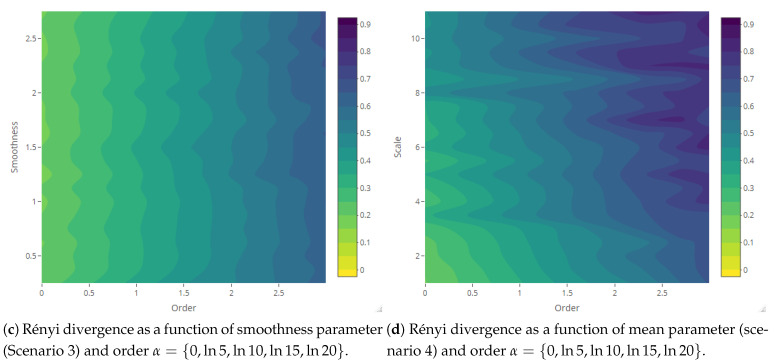
Rényi divergence between point point patterns and random fields as a function of variance, mean, smoothness and scale parameters (Scenarios 1–4) and order α={log0,log5,log10,log15,log20}.

**Figure 21:**
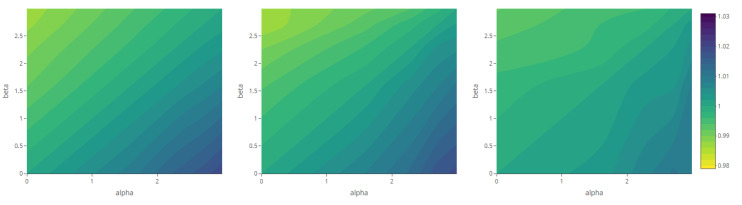
Generalized relative complexity as a function of the variance parameter, for $\sigma^{2}=0$ (**left**), $\sigma^{2}=2.5$ (**center**) and $\sigma^{2}=5$ (**right**) and order α,β∈{0,0.6,1.2,1.8,2.4} (fixed parameters as in Scenario 1).

**Figure 22:**
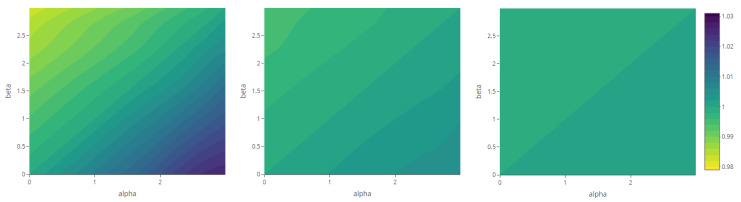
Generalized relative complexity as a function of the mean parameter μ=0 (**left**), μ=2 (**center**) and μ=4 (**right**) and order α,β∈{0,0.6,1.2,1.8,2.4} (fixed parameters as in Scenario 2).

**Figure 23:**
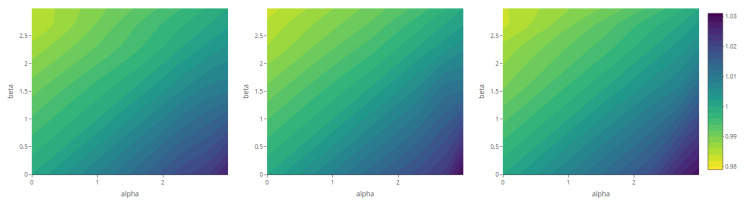
Generalized relative complexity as a function of the smoothness parameter ν=0 (**left**), ν=2.5 (**center**) and ν=5 (**right**) and order α,β∈{0,0.6,1.2,1.8,2.4} (fixed parameters as in Scenario 3).

**Figure 24:**
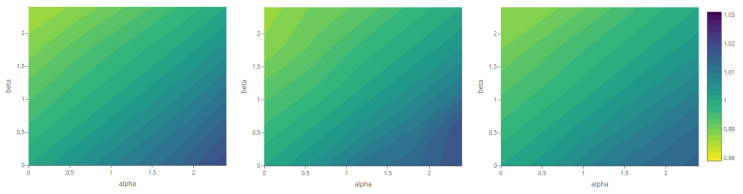
Generalized relative complexity as a function of the scale parameter ρ=1 (**left**), ρ=5 (**center**) and ρ=11 (**right**) and order α,β∈{0,0.6,1.2,1.8,2.4} (fixed parameters as in Scenario 4).

The maps representing the Rényi divergences in Figure 20 allow us to visualize how the distortion induced by the deformation parameter is reflected in the structural local departure of the point patterns from the intensity fields. Similarly, those referring to the generalized relative complexity show the sensitivity of Rényi divergences with respect to incremental changes in the deformation parameter (Figure 21, Figure 22, Figure 23 and Figure 24).

## 5. Discussion

As a result, from the study performed, it can be emphasized that the information transfer and, in general, the structure of the processes are mainly determined by the values of the mean, variance and scale parameters. Conversely, the value of the smoothness parameter does not have a perceptible effect on the structural information transferred between the phases.

In particular, among other aspects, the analysis shows a significant increase in the system complexity and a loss of diversity for large magnitudes of the variance parameter (both marginally, for the intensity field and point pattern). Regarding the local coherence measured in terms of divergence, we can observe an enlargement of the structural information transferred as a result of the increase in the mean and variance parameter values. On the other hand, the increase in the scale parameter results in the loss of structure of the intensity field, raising the stochasticity inherent to the pattern generation, with an associated increase in the divergence between both phases.

## 6. Conclusions

An assessment focused on the relevance of using entropy, divergence and complexity measures for the evaluation of the global and local structural information transfer from the intensity fields to the point patterns of log-Gaussian Cox processes is presented. Maps of generalized ordinary and relative information complexity measures are derived, showing the sensitivity of the distributions involved in relation to both stochasticity phases, with respect to the deformation parameter under different scenarios.

In general terms, and depending on the specific case, the transfer of structural information from the intensities to the subsequent point patterns is quantified by the information and complexity estimates, reflecting as well the contribution of the additional source of variability involved in the second step.

Among other relevant lines for continuing research, the study performed motivates the subsequent formal investigation of several analytical aspects involved in the structural complexity of log-Gaussian Cox processes. Multifractal Cox processes [29] constitute an important extension for analysis based on the connection between information complexity measures and multifractal dimensions [16]. In the spatiotemporal context, implications with reference to predictive risk evaluation and mapping [30], regarding the information complexity characterization of different scenarios, are also under development by the authors.

A further insight to be considered in future research is quantifying the information transfer from individual external covariates and providing an inferential framework to attach significance to this sort of statistical testing. In this paper, we have been restricted to a descriptive analysis, but the upgrade into the inferential context offers a natural motivation for the continuation of the study presented. Future developments also include using and comparing alternative models under the information complexity approach. The development of a model classification in terms of these measures would be worth exploring.

## Figures and Tables

**Table 1:** Parameter configurations for the scenarios considered in the analysis.

	Variable Parameters	Fixed Parameters		
Scenario 1	$\sigma =\left[0,0.25,\ldots ,5\right]$	ν=1	ρ=1	μ=0
Scenario 2	$\mu =\left[0,0.2,\ldots ,4\right]$	σ=1	ν=1	ρ=1
Scenario 3	$\nu =\left[0.25,0.375,\ldots ,2.75\right]$	σ=1	ρ=1	μ=0
Scenario 4	$\rho =\left[1,1.5,\ldots ,11\right]$	σ=1	ν=1	μ=0

**Table 2:** Table of ‘Inter-Intra’ standard deviations of Shannon entropy for point patterns generated under Scenario 1, with multiple and single replicates.

Parameter Value	Shannon Entropy SD			
	Multiple Patterns			Single Pattern
	Mean Intra SD	Inter-Mean SD	Total SD	Inter-Single SD
$\sigma^{2}=0$	0.0782	0.0076	0.0784	0.0820
$\sigma^{2}=0.25$	0.0765	0.0552	0.0943	0.0914
$\sigma^{2}=0.5$	0.0737	0.0607	0.0954	0.0911
$\sigma^{2}=0.75$	0.0749	0.0849	0.1133	0.1245
$\sigma^{2}=1$	0.0733	0.0966	0.1211	0.1279
$\sigma^{2}=1.25$	0.0743	0.1127	0.1349	0.1364
$\sigma^{2}=1.5$	0.0709	0.1497	0.1654	0.2081
$\sigma^{2}=1.75$	0.0702	0.1590	0.1734	0.1767
$\sigma^{2}=2$	0.0685	0.1432	0.1584	0.2025
$\sigma^{2}=2.25$	0.0695	0.1856	0.1977	0.2141
$\sigma^{2}=2.5$	0.0650	0.2227	0.2312	0.2758
$\sigma^{2}=2.75$	0.0619	0.2187	0.2266	0.3048
$\sigma^{2}=3$	0.0607	0.3478	0.3516	0.2188
$\sigma^{2}=3.25$	0.0625	0.2711	0.2774	0.2982
$\sigma^{2}=3.5$	0.0574	0.3561	0.3591	0.2585
$\sigma^{2}=3.75$	0.0560	0.2575	0.2627	0.2949
$\sigma^{2}=4$	0.0516	0.3702	0.3722	0.3422
$\sigma^{2}=4.25$	0.0519	0.3046	0.3079	0.4179
$\sigma^{2}=4.5$	0.0495	0.4012	0.4025	0.3855
$\sigma^{2}=4.75$	0.0493	0.3999	0.4013	0.3825
$\sigma^{2}=5$	0.0471	0.3920	0.3131	0.4212

**Table 3:** Table of ‘Inter-Intra’ standard deviations of Kullback–Leibler divergence for point patterns generated under Scenario 1, with multiple and single replicates.

Parameter Value	Kullback–Leibler Divergence SD			
	Multiple Patterns			Single Pattern
	Mean Intra SD	Inter-Mean SD	Total SD	Inter-Single SD
$\sigma^{2}=0$	0.0782	0.0076	0.0784	0.0820
$\sigma^{2}=0.25$	0.0698	0.0561	0.0895	0.0911
$\sigma^{2}=0.5$	0.0600	0.0707	0.0927	0.0912
$\sigma^{2}=0.75$	0.0532	0.0717	0.0894	0.0892
$\sigma^{2}=1$	0.0469	0.0655	0.0807	0.1051
$\sigma^{2}=1.25$	0.0429	0.0806	0.0917	0.1064
$\sigma^{2}=1.5$	0.0358	0.0778	0.0860	0.0865
$\sigma^{2}=1.75$	0.0326	0.0749	0.0820	0.0767
$\sigma^{2}=2$	0.0290	0.0666	0.0729	0.0914
$\sigma^{2}=2.25$	0.0282	0.0812	0.0863	0.0686
$\sigma^{2}=2.5$	0.0249	0.0788	0.0830	0.0759
$\sigma^{2}=2.75$	0.0201	0.0716	0.0746	0.0696
$\sigma^{2}=3$	0.0184	0.0632	0.0661	0.0649
$\sigma^{2}=3.25$	0.0177	0.0598	0.0626	0.0638
$\sigma^{2}=3.5$	0.0150	0.0491	0.0516	0.0512
$\sigma^{2}=3.75$	0.0143	0.0530	0.0551	0.0574
$\sigma^{2}=4$	0.0117	0.0493	0.0510	0.0572
$\sigma^{2}=4.25$	0.0116	0.0490	0.0505	0.0575
$\sigma^{2}=4.5$	0.0100	0.0370	0.0384	0.0567
$\sigma^{2}=4.75$	0.0103	0.0543	0.0556	0.0387
$\sigma^{2}=5$	0.0088	0.0384	0.0396	0.0428
